# Utility of PSMA PET/CT in Staging and Restaging of Renal Cell Carcinoma: A Systematic Review and Metaanalysis

**DOI:** 10.2967/jnumed.124.267417

**Published:** 2024-07

**Authors:** Moe S. Sadaghiani, Saradha Baskaran, Michael A. Gorin, Steven P. Rowe, Jean-Claude Provost, Iryna Teslenko, Roman Bilyk, Hong An, Sara Sheikhbahaei

**Affiliations:** 1 Russell H. Morgan Department of Radiology and Radiological Science, Johns Hopkins University School of Medicine, Baltimore, Maryland;; 2 Lantheus, Bedford, Massachusetts;; 3 Milton and Carroll Petrie Department of Urology, Icahn School of Medicine at Mount Sinai, New York, New York; and; 4 Department of Radiology, University of North Carolina School of Medicine, Chapel Hill, North Carolina

**Keywords:** renal cell carcinoma, prostate-specific membrane antigen, PSMA PET/CT, metaanalysis

## Abstract

Prostate-specific membrane antigen (PSMA) is expressed in the neovasculature of multiple solid tumors, including renal cell carcinoma (RCC). Studies have demonstrated promising results on the utility of PSMA-targeted PET/CT imaging in RCC. This report aims to provide a systematic review and metaanalysis on the utility and detection rate of PSMA PET/CT imaging in staging or evaluation of primary RCC and restaging of metastatic or recurrent RCC. **Methods:** Searches were performed in PubMed, Embase, and abstract proceedings (last updated, August 2023). Studies that provided a lesion-level detection rate of PSMA radiotracers in staging or restaging of RCC were included in the metaanalysis. The overall pooled detection rate with a 95% CI was estimated, and subgroup analysis was performed when feasible. **Results:** Nine studies comprising 152 patients (133 clear cell RCC [ccRCC], 19 other RCC subtypes) were included in the metaanalysis. The pooled detection rate of PSMA PET/CT in evaluation of primary or metastatic RCC was estimated to be 0.83 (95% CI, 0.67–0.92). Subgroup analysis showed a pooled PSMA detection rate of 0.74 (95% CI, 0.57–0.86) in staging or evaluation of primary RCC lesions and 0.87 (95% CI, 0.73–0.95) in restaging of metastatic or recurrent RCC. Analysis based on the type of radiotracer showed a pooled detection rate of 0.85 (95% CI, 0.62–0.95) for ^68^Ga-based PSMA tracers and 0.92 (95% CI, 0.76–0.97) for ^18^F-DCFPyL PET/CT. Furthermore, in metastatic ccRCC, the available data support a significantly higher detection rate for ^18^F-DCFPyL PET/CT than for conventional imaging modalities (2 studies). **Conclusion:** Our preliminary results show that PSMA PET/CT could be a promising alternative imaging modality for evaluating RCC, particularly metastatic ccRCC. Large prospective studies are warranted to confirm clinical utility in the staging and restaging of RCC.

## Visual Abstract

**Figure:**
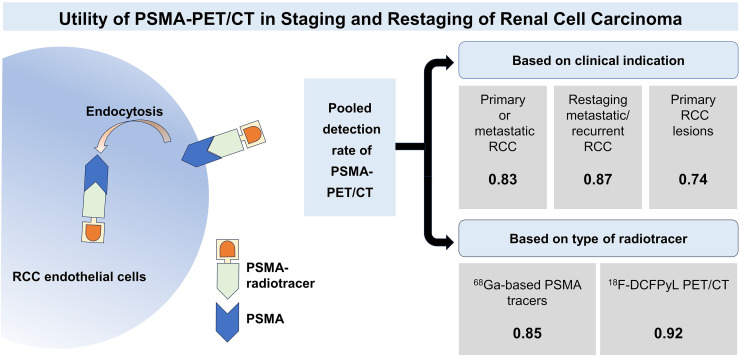

Renal cell carcinoma (RCC) accounts for 4% of the global cancer burden and 90% of all primary renal malignancies (1*,*^2^). RCC is the most lethal genitourinary cancer, as it often remains undetected during its early stages because of a lack of specific symptoms. Approximately 20%–30% of the patients present with metastases at initial diagnosis (2). Accurate staging and characterization of metastases are crucial for planning the treatment of patients with RCC. Conventional imaging techniques such as ultrasound, contrast-enhanced CT, MRI, and bone scintigraphy have been used for the diagnosis and staging of RCC (3*,*^4^). The current gold standard modality for evaluating metastatic disease in patients with inconclusive radiologic findings or for surveillance is contrast-enhanced CT using ^18^F-FDG PET/CT. However, this technique is not sensitive or comprehensive enough to detect early metastatic lesions and is associated with false-negative results, particularly in small lesions (e.g., <1 cm) or low-grade tumors (5). Approximately 25% of patients experience metastases after undergoing surgery for a seemingly resectable condition (6). Therefore, there is a need for a more sensitive modality for early detection and timely management of patients with metastatic RCC. Recent reports on the superiority of prostate-specific membrane antigen (PSMA)–targeted PET imaging over the conventional modalities for prostate cancer at initial staging and recurrence have increased interest in exploring its utility for RCC (7–9).

PSMA is a type II transmembrane glycoprotein highly expressed in prostate cancer cells, as well as in the endothelial cells within the neovasculature of multiple solid tumors, including RCC (10*,*^11^). Clear cell RCC (ccRCC) is the most common RCC subtype and is generally the most aggressive, although there are numerous other subtypes with varying aggressiveness, including chromophobe RCC and papillary RCC (12). PSMA expression varies greatly across RCC subtypes. PSMA is strongly expressed in ccRCC (76.2%–88%) compared with chromophobe RCC (31.2%–60%) and is rarely detectable in papillary RCC (13–15). With high levels of neovascularity and increased PSMA expression, patients with ccRCC are potential candidates for PSMA PET/CT.

The most widely used PSMA-targeted PET imaging probes are ^68^Ga- and ^18^F-labeled. Compared with ^68^Ga,^18^F has a longer half-life and higher target-to-background resolution (16). Multiple preliminary studies have investigated the clinical utility of PSMA PET/CT in RCC using different PSMA-directed radiotracers and shown promising results (17–21). Reviews on the role of PSMA PET/CT in the evaluation and management of RCC have also been published (22–24). However, there is a lack of metaanalysis in the available literature that would give better insight into the role of PSMA PET/CT in assessing RCC. In this report, we have provided a systematic review on the utility of PSMA PET/CT in staging and restaging of RCC and performed a metaanalysis on the detection rate of PSMA PET/CT in staging or evaluation of primary RCC and restaging of metastatic or recurrent RCC.

## MATERIALS AND METHODS

### Search Strategy

A systematic literature review was conducted on August 25, 2023, according to the Preferred Reporting Items for Systematic Review and Meta-Analyses (PRISMA) guidelines (25). The search was performed in PubMed, Embase, and abstract proceedings of major scientific meetings (Society of Nuclear Medicine and Molecular Imaging, European Association of Nuclear Medicine) to identify relevant published studies without any restrictions on language, publication date, or publication status. The search strategy was based on the following combination of keywords: (A) “renal cell carcinoma” OR “RCC” AND (B) “PSMA” OR “prostate-specific membrane antigen.” Institutional review board approval was not required since it was a retrospective analysis of previously published studies.

### Criteria for Study Consideration

Clinical studies investigating the utility of PSMA PET/CT imaging in staging or restaging of patients with RCC (ccRCC or non-ccRCC) were included. Index tests included ^18^F-DCFPyL, ^18^F-PSMA-1007, ^68^Ga-PSMA-11, or ^68^Ga-P16–093 PET/CT scans. The inclusion criteria included all studies that provided the lesion-based detection rate for any PSMA radiotracers in patients with RCC.

### Selection of Studies, Data Extraction, and Study Outcome

All records identified through the electronic search were initially screened for eligibility based on the title and abstract. Two of the authors performed this screening, which excluded review articles, editorials, and irrelevant citations. The full texts of the potentially relevant publications were retrieved and independently checked by the 2 authors for predefined inclusion criteria.

The 2 authors independently extracted the following data from each included study: bibliographic details, patient demographics and disease characteristics, index tests, number of patients, tumor histopathology, and detection rates. The overall pooled detection rate with 95% CIs was estimated among all included studies. In addition, subgroup analysis was performed to estimate the detection rate of ^18^F-DCFPyL and ^68^Ga-PSMA PET/CT in patients with metastatic RCC and to compare the performance of PSMA PET/CT relative to other conventional imaging modalities, when feasible (only 2 studies).

### Statistical Analysis and Data Synthesis

A lesion-based metaanalysis of single proportions was performed to calculate the pooled detection rate of PSMA PET/CT in patients with RCC using meta package (version 6.5-0) in R version 4.3.1. Forest plots of detection rates were created to display variations in the results of the individual studies. Logit transformation with the inverse variance method was used to perform a metaanalysis of proportions. The *I*^2^ index was calculated to quantify heterogeneity. *I*^2^ lies between 0% and 100%, with respective values of approximately 25%, 50%, and 75% indicating low, moderate, and high heterogeneity. To deal with heterogeneity, random-effect assumptions were used for synthesizing metaanalytic data (26). Funnel plots were used to assess publication bias.

## RESULTS

### Search Results and Study Characteristics

**FIGURE 1.**
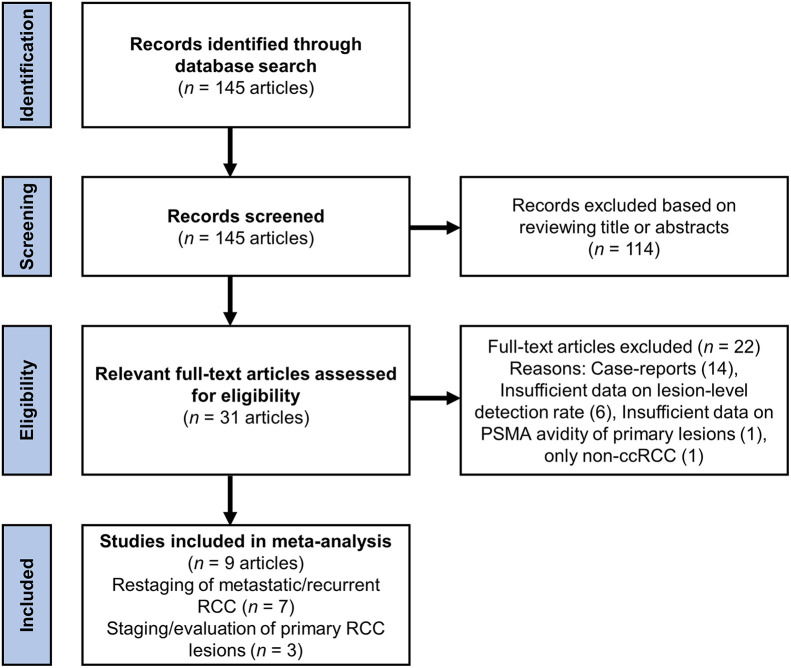
Flowchart of systematic review.

Using the comprehensive search strategy outlined in the methods section, we identified 145 articles, of which 114 were excluded by initial screening of title and abstract. The full texts of the remaining 31 studies were reviewed, and 22 studies were excluded. In total, 9 articles were included in the final metaanalysis and quantitative synthesis (Fig. 1).

Supplemental Tables 1 and 2 (16–21*,*^27^–^51^) summarize the published literature on the utility of PET/CT imaging using different PSMA-directed radiotracers, including 12 articles on ^18^F-based tracers (^18^F-DCFPyL, ^18^F-PSMA-1007) and 19 articles on ^68^Ga-based tracers (^68^Ga-PSMA-11, ^68^Ga-P16–093), in patients with RCC (31 studies, including case reports and case series) (supplemental materials are available at http://jnm.snmjournals.org). In these studies, PSMA PET/CT was performed for staging and restaging of RCC, evaluation of primary RCC lesions, or other purposes (e.g., prostate cancer restaging) with incidental detection of RCC metastases.

### Detection Rate of PSMA PET/CT in Staging or Restaging of RCC (Lesion-Level Analysis)

#### All Studies

**FIGURE 2.**
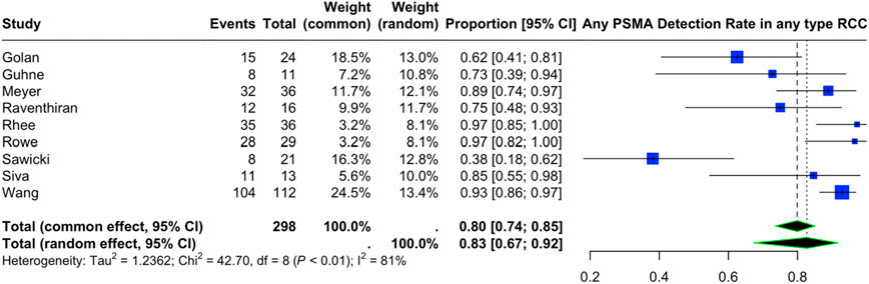
Lesion-based detection rate of PSMA PET/CT in staging and restaging of RCC (all included studies).

Nine articles, including 152 patients (133 ccRCC, 19 other RCC subtypes), provided information on the lesion-level detection rate of PSMA PET/CT performed for either staging and evaluation of primary RCC lesions or restaging of metastatic or recurrent RCC. The forest plot representing the pooled data from all included studies is depicted in Figure 2. The estimated pooled lesion-level detection rate of PET/CT with any PSMA radiotracer was 0.83 (95% CI, 0.67–0.92). There was high heterogeneity among the included studies (*I*^2^ = 81%).

Subgroup analysis was based on the clinical indication (restaging of metastatic RCC vs. staging or evaluation of primary RCC), histopathology (all subtypes of RCC vs. studies that included solely ccRCC), and type of radiotracer (^18^F- vs. ^68^Ga-based PSMA radiotracers), when feasible.

#### Restaging of Metastatic or Recurrent RCC

**FIGURE 3.**
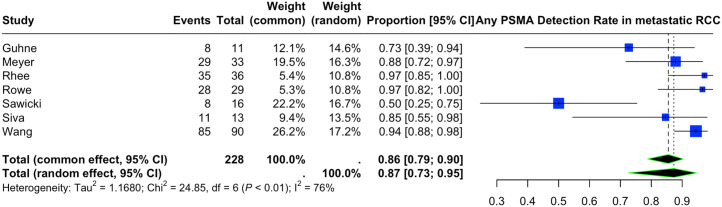
Lesion-based detection rate of PSMA PET/CT in restaging of patients with metastatic or recurrent RCC.

Seven articles, including 90 patients (87 ccRCC, 3 other RCC subtypes), provided information on the lesion-level detection rate of PSMA PET/CT in restaging of metastatic or recurrent RCC (Fig. 3). The estimated pooled lesion-level detection rate of any type of PSMA radiotracer was 0.87 (95% CI, 0.73–0.95). Limiting the cases to studies that included solely ccRCC pathology resulted in a pooled detection rate of 0.85 (95% CI, 0.64–0.95). There was substantial heterogeneity among the included studies, with an *I*^2^ of 76%.

The lowest detection rate has been reported by Sawicki et al. (50%), with all PET-negative metastases being subcentimeter pulmonary nodules in 1 patient (39). This study was identified as a possible source contributing to the high heterogeneity. Exclusion of this study from the analysis significantly improved the study heterogeneity (leave-one-out method), with a pooled detection rate of 0.91 (95% CI, 0.83–0.95; *I*^2^ = 42%) (Supplemental Fig. 1).

#### Staging or Evaluation of Primary RCC Lesions

**FIGURE 4.**
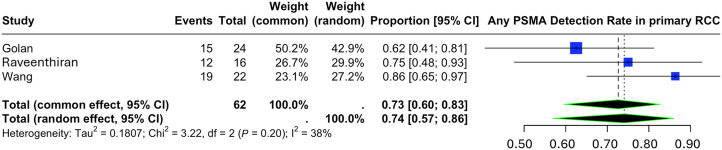
Detection rate of PSMA PET/CT in staging or evaluation of primary RCC.

Three studies, including 62 patients (48 ccRCC, 14 other subtypes), reported the lesion-level detection rate of ^68^Ga-PSMA PET/CT in the staging or evaluation of primary RCC lesions. Only malignant lesions and their PSMA PET positivity data were included in the analysis. The pooled detection rate of PSMA PET/CT for primary RCC was 0.74 (95% CI, 0.57–0.86), with an *I*^2^ of 38%. In other words, approximately 74% (46/62) of primary RCC lesions were PSMA-positive (Fig. 4).

#### ^68^Ga-Based Versus ^18^F-Based PSMA Radiotracer

**FIGURE 5.**
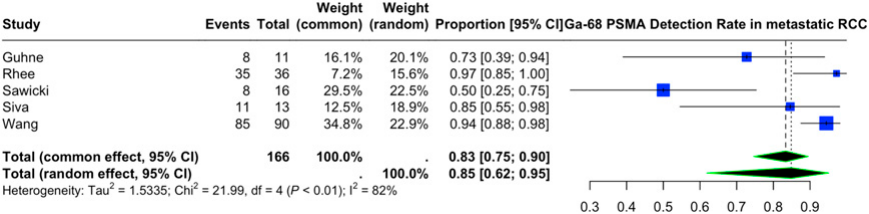
Lesion-based detection rate of ^68^Ga-based PSMA radiotracers in restaging of patients with metastatic RCC.

Among the included studies on patients with metastatic RCC, ^68^Ga-based PSMA radiotracers were used in 5 studies including 75 patients (72 ccRCC, 3 non-ccRCC). The pooled detection rate in these studies was estimated to be 0.85 (95% CI, 0.62–0.95; *I*^2^ = 82%) (Fig. 5). Limiting the analysis to studies that included solely ccRCC pathology resulted in a pooled detection rate of 0.80 (95% CI, 0.53–0.93).

**FIGURE 6.**
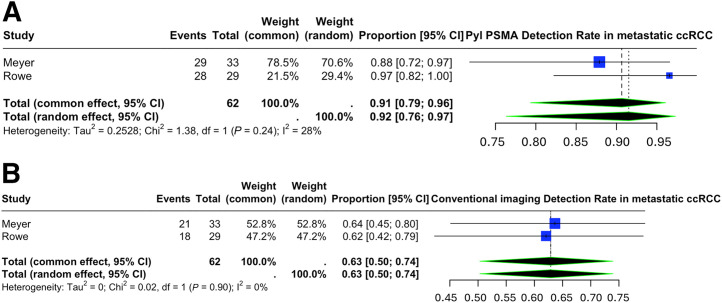
Lesion-based detection rate of ^18^F-DCFPyL PSMA in restaging of metastatic RCC (A), in comparison with conventional imaging modalities (B).

Two of the included studies on 19 patients with metastatic RCC (all ccRCC subtype) evaluated the utility of an ^18^F-based PSMA radiotracer (^18^F-DCFPyL) and provided a direct comparison with conventional imaging (17*,*^19^). Our analysis revealed that ^18^F-DCFPyL PET/CT provides a significantly higher detection rate in metastatic RCC than conventional imaging modalities such as CT and MRI, with pooled estimates of 0.92 (95% CI, 0.76–0.97) versus 0.63 (95% CI, 0.50–0.74), respectively (Fig. 6). There was low heterogeneity in this subgroup analysis (*I*^2^ = 28% and 0%, respectively).

### Publication Bias

**FIGURE 7.**
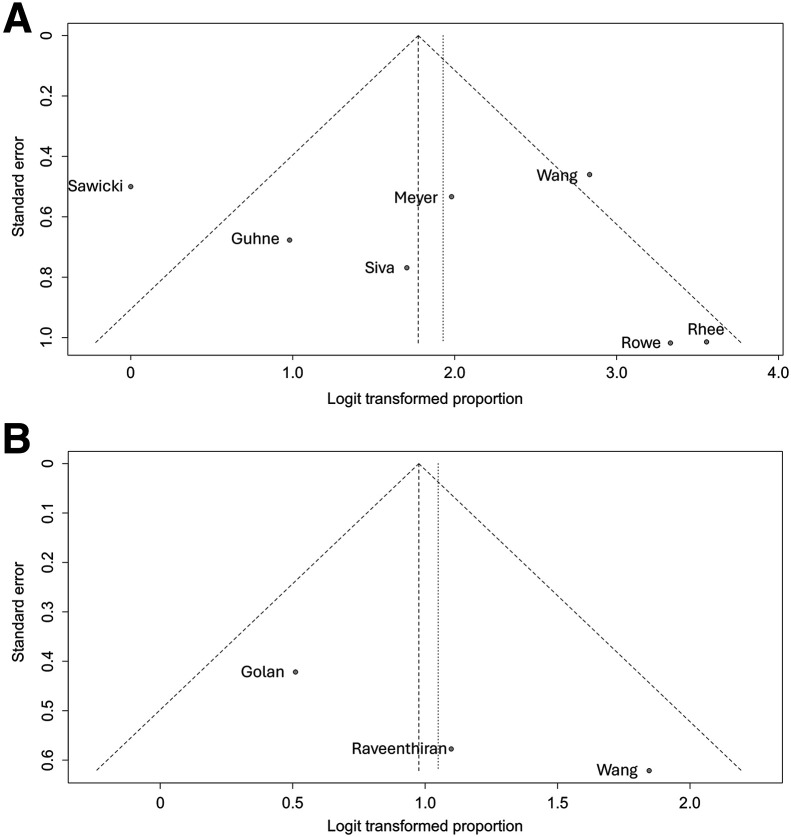
Funnel plot of publication bias on detection rate of PSMA PET/CT in restaging of metastatic RCC (A) and evaluation of primary lesions (B).

Qualitative evaluation using funnel plots revealed relatively symmetric plots suggestive of a low probability of publication bias (Fig. 7).

## DISCUSSION

In this study, we systematically reviewed all the available literature on the utility of PSMA PET/CT imaging in staging or restaging of RCC (supplemental tables) and performed a metaanalysis on the eligible relevant studies when feasible. To our knowledge, this was the first metaanalysis assessing the detection rate of PSMA PET/CT in this patient population. Our results revealed the potential role of PSMA PET/CT in the staging or evaluation of primary RCC lesions and in the restaging of metastatic or recurrent RCC, with a pooled detection rate of 0.74 (95% CI, 0.57–0.86) and 0.87 (95% CI, 0.73–0.95), respectively. Our subgroup analysis revealed that both ^68^Ga-based (7 studies) and ^18^F-based (2 studies) PSMA radiotracers have a high detection rate for evaluation of metastatic RCC (17–21*,*^36^*,*^37^*,*^39^*,*^41^).

We found high heterogeneity among the included studies (*I*^2^ > 75%). The heterogeneous study types, differences in the radiotracers used across studies, and mixed patient populations, including both ccRCC and non-ccRCC and different stages of the disease, could have contributed to the high heterogeneity. In Sawicki et al. (detection rate, 50%), all PET-negative metastases were subcentimeter pulmonary nodules in 1 patient (39). This study was identified as a source of heterogeneity. Pulmonary nodules are prone to have decreased tracer activity, which at least partially is explained by breathing-related motion degradation. Furthermore, the subcentimeter size of nodules limits PET resolution because of partial-volume effects, although with targeted radiotracers, it is possible to drive contrast resolution to overcome spatial resolution limitations. Redoing the metaanalysis after exclusion of this study resulted in improved heterogeneity (*I*^2^ = 42%). Primary versus metastatic disease status was identified as another source of heterogeneity, and subgroup analysis on this population improved the heterogeneity (*I*^2^ = 38%). In addition, most of those studies were conducted retrospectively and lacked pathologic proof of disease at the site of radiotracer uptake; this factor could have contributed to heterogeneity.

Demirci et al. (50) and Rowe et al. (17) were the first to report the utility of PSMA-targeted radiotracers for imaging patients with ccRCC using ^68^Ga-PSMA-11 and ^18^F-DCFPyL, respectively. Subsequently, multiple small-scale preliminary studies demonstrated promising results for PSMA PET/CT in staging and restaging of RCC, with the main advantage being the possibility of detecting distant metastasis (23). Patients with metastatic RCC have a poor prognosis, with a 5-y survival rate of only 12% (12). Therefore, accurate staging and assessment of metastases are crucial for managing patients and determining the treatment strategy. Siva et al. (41), Raveenthiran et al. (20), and Guhne et al. (37) examined the diagnostic utility of ^68^Ga-PSMA PET/CT for evaluating disease extent in patients with metastatic RCC. In a retrospective series of 8 patients with oligometastatic RCC, Siva et al. demonstrated higher uptake on ^68^Ga-PSMA than on ^18^F-FDG PET, suggesting ^68^Ga-PSMA PET to be a more sensitive modality than ^18^F-FDG PET for diagnostic evaluation of metastatic RCC (41). Raveenthiran et al. examined the effectiveness of ^68^Ga-PSMA PET/CT, compared with conventional imaging, in guiding management decisions (20).

ccRCC accounts for over 75% of RCC diagnoses (12) and has strong PSMA expression relative to other RCC subtypes (13). A retrospective case series of 38 patients with ^68^Ga-PSMA PET/CT for staging or restaging of RCC revealed the strongest detection rate in patients with ccRCC and a clinical management change in 43.8% of primary staging cases and 40.9% of restaging cases (20). Guhne et al. demonstrated molecular PSMA expression in all PET-positive lesions, with no correlation between histopathologic findings (extent and intensity of PSMA expression) and PET/CT parameters (SUV_max_, SUV_mean_, lesion-to-muscle ratio) in patients with metastatic ccRCC; however, this lack of correlation was because most lung metastases showed low tracer uptake (37). Seven of our included studies evaluated the role of PSMA PET/CT in patients with metastatic or recurrent RCC, with a pooled detection rate of 87%. Most included patients in these studies had ccRCC pathology (87 ccRCC, 3 other subtypes). In addition, our subgroup analysis on studies that included only ccRCC patients did not show a significant change in the detection rate of PSMA PET/CT. Thus, the presence of a different tumor histopathology is not likely to significantly affect our results in this analysis.

Two of the included studies provided a direct comparison of PSMA PET/CT (^18^F-DCFPyL) and conventional imaging in metastatic ccRCC and support a higher detection rate of PSMA PET/CT, with a pooled detection rate of 0.92 (95% CI, 0.76–0.97) versus 0.63 (95% CI, 0.50–0.74), respectively (17*,*^19^). Rowe et al. prospectively analyzed 5 patients with metastatic ccRCC and identified 29 lesions on at least one modality. Of these, 18 metastatic lesions were identified on conventional imaging, whereas 28 sites were identified on ^18^F-DCFPyL PET/CT, 17 of which corresponded to the disease site seen on conventional imaging. The study reported a higher detection rate (97% vs. 62%) and higher sensitivity (94.7% vs. 78.9%) for ^18^F-DCFPyL PET/CT than for conventional imaging in the detection of metastatic lesions (17). Meyer et al. conducted a prospective study to evaluate the clinical utility of ^18^F-DCFPyL PET/CT in patients with presumed oligometastatic ccRCC based on conventional imaging. In total, 33 metastatic sites of disease were identified in 17 oligometastatic ccRCC patients, of which 29 sites were detected on ^18^F-DCFPyL PET/CT and 21 metastatic lesions were identified on conventional imaging. Seventeen of 21 (81%) metastatic lesions detected on conventional imaging had radiotracer uptake. In 4 patients (28.6%), 12 lesions not detected on conventional imaging were identified on ^18^F-DCFPyL PET/CT, and 3 of these patients were no longer considered oligometastatic. The detection rates of ^18^F-DCFPyL PET/CT and conventional imaging for identifying sites of metastatic disease were reported as 87.9% and 63.4%, respectively (19). Rhee et al. reported a sensitivity of 92% for ^68^Ga-PSMA PET/CT in detecting RCC metastatic lesions, compared with 68.6% for conventional CT (18). A recent study compared the utility of ^68^Ga-PSMA PET/CT in ccRCC and non-ccRCC and reported that ^68^Ga-PSMA PET/CT had accuracy and sensitivity superior to conventional imaging in the detection of metastatic lesions in ccRCC (40).

The major limitations of the current analysis are high heterogeneity among the studies, lack of definitive indications for the application of PSMA PET/CT in RCC patients, the small number of patients analyzed, lack of data on the location and size of RCC lesions, and the retrospective nature of most of the studies included in the analysis. Despite these limitations, our preliminary results shed light on the potential role of PSMA PET/CT in the detection and characterization of metastatic RCC. Large prospective trials with robust inclusion criteria and pathologic confirmation of lesions would be of value to validate the diagnostic efficiency of PSMA PET/CT in RCC, particularly in patients at high risk for metastatic disease at initial staging, response assessment, or surveillance monitoring; in patients with oligometastatic disease; and in patients who can potentially be considered for future radioligand PSMA-targeted therapy.

## CONCLUSION

Our metaanalysis showed the detection potential of PSMA PET/CT in staging primary RCC lesions and restaging metastatic or recurrent RCC. Although our findings are based on small-scale studies with high heterogeneity, the preliminary results suggest merit in the use of PSMA PET/CT in RCC, particularly when performed for restaging of metastatic or recurrent disease.

## DISCLOSURE

Research funding was provided by Lantheus. No other potential conflict of interest relevant to this article was reported.

## Notes

KEY POINTS **QUESTION:** Does PSMA PET/CT have the potential to be an effective alternative imaging modality for patients with RCC? **PERTINENT FINDINGS:** This metaanalysis demonstrated high detection rates for PSMA-targeted imaging of RCC using both ^18^F- and ^68^Ga-labeled agents. **IMPLICATIONS FOR PATIENT CARE:** These findings suggest a new opportunity for improved detection of metastatic RCC using PSMA-targeted imaging agents.

## References

[1] Siegel RL Miller KD Fuchs HE Jemal A. Cancer statistics, 2021. CA Cancer J Clin. 2021;71:7–33. 33433946 10.3322/caac.21654

[2] Ljungberg B Campbell SC Choi HY. The epidemiology of renal cell carcinoma. Eur Urol. 2011;60:615–621. 21741761 10.1016/j.eururo.2011.06.049

[3] Leveridge MJ Bostrom PJ Koulouris G Finelli A Lawrentschuk N. Imaging renal cell carcinoma with ultrasonography, CT and MRI. Nat Rev Urol. 2010;7:311–325. 20479778 10.1038/nrurol.2010.63

[4] Rossi SH Prezzi D Kelly-Morland C Goh V. Imaging for the diagnosis and response assessment of renal tumours. World J Urol. 2018;36:1927–1942. 29948048 10.1007/s00345-018-2342-3 PMC6280818

[5] Brufau BP Cerqueda CS Villalba LB Izquierdo RS Gonzalez BM Molina CN. Metastatic renal cell carcinoma: radiologic findings and assessment of response to targeted antiangiogenic therapy by using multidetector CT. Radiographics. 2013;33:1691–1716. 24108558 10.1148/rg.336125110

[6] Psutka SP Master VA. Role of metastasis-directed treatment in kidney cancer. Cancer. 2018;124:3641–3655. 29689599 10.1002/cncr.31341

[7] Tanaka T Yang M Froemming AT. Current imaging techniques for and imaging spectrum of prostate cancer recurrence and metastasis: a pictorial review. Radiographics. 2020;40:709–726. 32196428 10.1148/rg.2020190121

[8] Pienta KJ Gorin MA Rowe SP. A phase 2/3 prospective multicenter study of the diagnostic accuracy of prostate specific membrane antigen PET/CT with ^18^F-DCFPyL in prostate cancer patients (OSPREY). J Urol. 2021;206:52–61. 33634707 10.1097/JU.0000000000001698 PMC8556578

[9] Morris MJ Rowe SP Gorin MA. Diagnostic performance of ^18^F-DCFPyL-PET/CT in men with biochemically recurrent prostate cancer: results from the CONDOR phase III, multicenter study. Clin Cancer Res. 2021;27:3674–3682. 33622706 10.1158/1078-0432.CCR-20-4573 PMC8382991

[10] Van de Wiele C Sathekge M de Spiegeleer B. PSMA expression on neovasculature of solid tumors. Histol Histopathol. 2020;35:919–927. 32282924 10.14670/HH-18-215

[11] Chang SS Reuter VE Heston WD Gaudin PB. Metastatic renal cell carcinoma neovasculature expresses prostate-specific membrane antigen. Urology. 2001;57:801–805. 11306418 10.1016/s0090-4295(00)01094-3

[12] Tung I Sahu A. Immune checkpoint inhibitor in first-line treatment of metastatic renal cell carcinoma: a review of current evidence and future directions. Front Oncol. 2021;11:707214. 34527581 10.3389/fonc.2021.707214 PMC8435744

[13] Baccala A Sercia L Li J Heston W Zhou M. Expression of prostate-specific membrane antigen in tumor-associated neovasculature of renal neoplasms. Urology. 2007;70:385–390. 17826525 10.1016/j.urology.2007.03.025

[14] Al-Ahmadie HA Olgac S Gregor PD. Expression of prostate-specific membrane antigen in renal cortical tumors. Mod Pathol. 2008;21:727–732. 18344976 10.1038/modpathol.2008.42

[15] Baraban EG Ged Y Singla N. Vascular expression of prostate-specific membrane antigen (PSMA) in MiTF family translocation renal cell carcinoma and related neoplasms. Appl Immunohistochem Mol Morphol. 2023;31:544–549. 37471632 10.1097/PAI.0000000000001142

[16] Gorin MA Rowe SP Hooper JE. PSMA-targeted ^18^F-DCFPyL PET/CT imaging of clear cell renal cell carcinoma: results from a rapid autopsy. Eur Urol. 2017;71:145–146. 27363386 10.1016/j.eururo.2016.06.019 PMC5516900

[17] Rowe SP Gorin MA Hammers HJ. Imaging of metastatic clear cell renal cell carcinoma with PSMA-targeted ^18^F-DCFPyL PET/CT. Ann Nucl Med. 2015;29:877–882. 26286635 10.1007/s12149-015-1017-z PMC4666821

[18] Rhee H Blazak J Tham CM. Pilot study: use of gallium-68 PSMA PET for detection of metastatic lesions in patients with renal tumour. EJNMMI Res. 2016;6:76. 27771904 10.1186/s13550-016-0231-6 PMC5075321

[19] Meyer AR Carducci MA Denmeade SR. Improved identification of patients with oligometastatic clear cell renal cell carcinoma with PSMA-targeted ^18^F-DCFPyL PET/CT. Ann Nucl Med. 2019;33:617–623. 31147927 10.1007/s12149-019-01371-8 PMC9774684

[20] Raveenthiran S Esler R Yaxley J Kyle S. The use of ^68^Ga-PET/CT PSMA in the staging of primary and suspected recurrent renal cell carcinoma. Eur J Nucl Med Mol Imaging. 2019;46:2280–2288. 31332498 10.1007/s00259-019-04432-2

[21] Wang G Li L Wang J. Head-to-head comparison of [^68^Ga]Ga-P16-093 and 2-[^18^F]FDG PET/CT in patients with clear cell renal cell carcinoma: a pilot study. Eur J Nucl Med Mol Imaging. 2023;50:1499–1509. 36600099 10.1007/s00259-022-06101-3

[22] Ahn T Roberts MJ Abduljabar A. A review of prostate-specific membrane antigen (PSMA) positron emission tomography (PET) in renal cell carcinoma (RCC). Mol Imaging Biol. 2019;21:799–807. 30617728 10.1007/s11307-018-01307-0

[23] Evangelista L Basso U Maruzzo M Novara G. The role of radiolabeled prostate-specific membrane antigen positron emission tomography/computed tomography for the evaluation of renal cancer. Eur Urol Focus. 2020;6:146–150. 30120074 10.1016/j.euf.2018.08.004

[24] Urso L Castello A Rocca GC. Role of PSMA-ligands imaging in renal cell carcinoma management: current status and future perspectives. J Cancer Res Clin Oncol. 2022;148:1299–1311. 35217902 10.1007/s00432-022-03958-7 PMC9114025

[25] Page MJ McKenzie JE Bossuyt PM. The PRISMA 2020 statement: an updated guideline for reporting systematic reviews. BMJ. 2021;372:n71. 33782057 10.1136/bmj.n71 PMC8005924

[26] DerSimonian R Laird N. Meta-analysis in clinical trials. Control Clin Trials. 1986;7:177–188. 3802833 10.1016/0197-2456(86)90046-2

[27] Rowe SP Gorin MA Hammers HJ Pomper MG Allaf ME Javadi MS. Detection of ^18^F-FDG PET/CT occult lesions with ^18^F-DCFPyL PET/CT in a patient with metastatic renal cell carcinoma. Clin Nucl Med. 2016;41:83–85. 26402128 10.1097/RLU.0000000000000995 PMC4834697

[28] Yin Y Campbell SP Markowski MC. Inconsistent detection of sites of metastatic non-clear cell renal cell carcinoma with PSMA-targeted [^18^F]DCFPyL PET/CT. Mol Imaging Biol. 2019;21:567–573. 30218388 10.1007/s11307-018-1271-2 PMC9774683

[29] Currie GM Trifunovic M Liu J Kim S Gurney H. ^18^F-DCFPyL PET/CT in metastatic renal cell carcinoma. J Nucl Med Technol. 2022;50:282–285. 34750233 10.2967/jnmt.121.262799

[30] Perry E Talwar A Sharma S. Non-prostate cancer tumours: incidence on ^18^F-DCFPyL PSMA PET/CT and uptake characteristics in 1445 patients. Eur J Nucl Med Mol Imaging. 2022;49:3277–3288. 35254481 10.1007/s00259-022-05721-z PMC9250467

[31] Zeng Y Luo J Liao H Chen P. Utility of ^18^F-prostate-specific membrane antigen 1007 in imaging of tumor thrombus of renal cell carcinoma. Clin Nucl Med. 2021;46:697–699. 33883493 10.1097/RLU.0000000000003664

[32] Marafi F Sasikumar A Al-Terki A Alfeeli M. ^18^F-PSMA 1007 in suspected renal cell carcinoma. Clin Nucl Med. 2020;45:377–378. 32209880 10.1097/RLU.0000000000003002

[33] Sadeq A Usmani S Esmail AA Fathallah W Alfeeli MA Marafi F. Incremental value of ^18^F-PSMA-1007 PET/CT in detection of metastatic renal cell carcinoma to the brain. Clin Nucl Med. 2022;47:627–628. 35675136 10.1097/RLU.0000000000004162

[34] Marafi F Sasikumar A Aldaas M Esmail A. ^18^F-PSMA-1007 PET/CT for initial staging of renal cell carcinoma in an end-stage renal disease patient. Clin Nucl Med. 2021;46:e65–e67. 33181733 10.1097/RLU.0000000000003354

[35] Xiong M Zhang W Zhou C Bao J Zang S Lin X. Application of ^18^F prostate-specific membrane antigen positron emission tomography/computed tomography in monitoring gastric metastasis and cancer thrombi from renal cell carcinoma. J Oncol. 2022;2022:5681463. 35154318 10.1155/2022/5681463 PMC8837453

[36] Golan S Aviv T Groshar D. Dynamic ^68^Ga-PSMA-11 PET/CT for the primary evaluation of localized renal mass: a prospective study. J Nucl Med. 2021;62:773–778. 33097628 10.2967/jnumed.120.251272

[37] Guhne F Seifert P Theis B Steinert M Freesmeyer M Drescher R. PSMA-PET/CT in patients with recurrent clear cell renal cell carcinoma: histopathological correlations of imaging findings. Diagnostics (Basel). 2021;11:1142. 34201583 10.3390/diagnostics11071142 PMC8304877

[38] Seront E Lhommel R Tombal B. Case report: early ^68^Ga-PSMA-PET metabolic assessment and response to systemic treatment for first-line metastatic clear cell renal cell carcinoma; about two clinical cases. Front Oncol. 2021;11:782166. 34950588 10.3389/fonc.2021.782166 PMC8689125

[39] Sawicki LM Buchbender C Boos J. Diagnostic potential of PET/CT using a ^68^Ga-labelled prostate-specific membrane antigen ligand in whole-body staging of renal cell carcinoma: initial experience. Eur J Nucl Med Mol Imaging. 2017;44:102–107. 26996777 10.1007/s00259-016-3360-2

[40] Li Y Zheng R Zhang Y. Special issue “The advance of solid tumor research in China”: ^68^Ga-PSMA-11 PET/CT for evaluating primary and metastatic lesions in different histological subtypes of renal cell carcinoma. Int J Cancer. 2023;152:42–50. 35751420 10.1002/ijc.34189 PMC9796964

[41] Siva S Callahan J Pryor D Martin J Lawrentschuk N Hofman MS. Utility of ^68^Ga prostate specific membrane antigen – positron emission tomography in diagnosis and response assessment of recurrent renal cell carcinoma. J Med Imaging Radiat Oncol. 2017;61:372–378. 28116853 10.1111/1754-9485.12590

[42] Meng L Zhang S Gao J. [^68^Ga]Ga-PSMA-11 PET/CT has potential application in predicting tumor HIF-2α expression and therapeutic response to HIF-2α antagonists in patients with RCC. Eur Radiol. 2022;32:6545–6553. 35357538 10.1007/s00330-022-08738-y

[43] Jha S Hemrom A Shamim SA Barwad A Batra A. ^68^Ga-PSMA PET/CT detecting metastatic lesion of RCC: missed on ^18^F-FDG PET/CT. Clin Nucl Med. 2023;48:e294–e296. 37133514 10.1097/RLU.0000000000004648

[44] Gao J Meng L Xu Q. ^68^Ga-PSMA-11 PET/CT parameter correlates with pathological VEGFR-2/PDGFR-beta expression in renal cell carcinoma patients. Mol Imaging Biol. 2022;24:759–768. 35451707 10.1007/s11307-022-01725-1

[45] Tariq A McGeorge S Pearce A. Characterization of tumor thrombus in renal cell carcinoma with prostate specific membrane antigen (PSMA) positron emission tomography (PET)/computed tomography (CT). Urol Oncol. 2022;40:276.e1–276.e9. 10.1016/j.urolonc.2022.03.007 35466037

[46] Udovicich C Callahan J Bressel M. Impact of prostate-specific membrane antigen positron emission tomography/computed tomography in the management of oligometastatic renal cell carcinoma. Eur Urol Open Sci. 2022;44:60–68. 36185587 10.1016/j.euros.2022.08.001 PMC9520507

[47] Filizoglu N Cetin IA Kissa TN Niftaliyeva K Ones T. ^68^Ga-PSMA PET/CT to distinguish brain metastasis of renal cell carcinoma from radiation necrosis after stereotactic radiosurgery. Clin Nucl Med. 2021;46:913–914. 34284481 10.1097/RLU.0000000000003820

[48] Zacho HD Nielsen JB Dettmann K Haberkorn U Petersen LJ. Incidental detection of thyroid metastases from renal cell carcinoma using ^68^Ga-PSMA PET/CT to assess prostate cancer recurrence. Clin Nucl Med. 2017;42:221–222. 28033223 10.1097/RLU.0000000000001522

[49] Saadat S Tie B Wood S Vela I Rhee H. Imaging tumour thrombus of clear cell renal cell carcinoma: FDG PET or PSMA PET? Direct in vivo comparison of two technologies. Urol Case Rep. 2017;16:4–5. 29034176 10.1016/j.eucr.2017.09.010 PMC5635239

[50] Demirci E Ocak M Kabasakal L. ^68^Ga-PSMA PET/CT imaging of metastatic clear cell renal cell carcinoma. Eur J Nucl Med Mol Imaging. 2014;41:1461–1462. 24756358 10.1007/s00259-014-2766-y

[51] Tariq A Kwok M Pearce A. The role of dual tracer PSMA and FDG PET/CT in renal cell carcinoma (RCC) compared to conventional imaging: a multi-institutional case series with intra-individual comparison. Urol Oncol. 2022;40:66.e1–66.e9. 10.1016/j.urolonc.2021.11.006 34895817

